# Convolutional neural network models for cancer type prediction based on gene expression

**DOI:** 10.1186/s12920-020-0677-2

**Published:** 2020-04-03

**Authors:** Milad Mostavi, Yu-Chiao Chiu, Yufei Huang, Yidong Chen

**Affiliations:** 1 Greehey Children’s Cancer Research Institute, University of Texas Health San Antonio, San Antonio, TX 78229 USA; 2 Department of Electrical and Computer Engineering, University of Texas at San Antonio, San Antonio, TX 78249 USA; 3 Department of Population Health Sciences, University of Texas Health San Antonio, San Antonio, TX 78229 USA

**Keywords:** Deep learning, Convolutional neural networks, The Cancer Genome Atlas, Cancer type prediction, Cancer gene markers, Breast cancer subtype prediction

## Abstract

**Background:**

Precise prediction of cancer types is vital for cancer diagnosis and therapy. Through a predictive model, important cancer marker genes can be inferred. Several studies have attempted to build machine learning models for this task however none has taken into consideration the effects of tissue of origin that can potentially bias the identification of cancer markers.

**Results:**

In this paper, we introduced several Convolutional Neural Network (CNN) models that take unstructured gene expression inputs to classify tumor and non-tumor samples into their designated cancer types or as normal. Based on different designs of gene embeddings and convolution schemes, we implemented three CNN models: 1D-CNN, 2D-Vanilla-CNN, and 2D-Hybrid-CNN. The models were trained and tested on gene expression profiles from combined 10,340 samples of 33 cancer types and 713 matched normal tissues of The Cancer Genome Atlas (TCGA). Our models achieved excellent prediction accuracies (93.9–95.0%) among 34 classes (33 cancers and normal). Furthermore, we interpreted one of the models, 1D-CNN model, with a guided saliency technique and identified a total of 2090 cancer markers (108 per class on average). The concordance of differential expression of these markers between the cancer type they represent and others is confirmed. In breast cancer, for instance, our model identified well-known markers, such as *GATA3* and *ESR1*. Finally, we extended the 1D-CNN model for the prediction of breast cancer subtypes and achieved an average accuracy of 88.42% among 5 subtypes. The codes can be found at https://github.com/chenlabgccri/CancerTypePrediction.

**Conclusions:**

Here we present novel CNN designs for accurate and simultaneous cancer/normal and cancer types prediction based on gene expression profiles, and unique model interpretation scheme to elucidate biologically relevance of cancer marker genes after eliminating the effects of tissue-of-origin. The proposed model has light hyperparameters to be trained and thus can be easily adapted to facilitate cancer diagnosis in the future.

## Background

Cancer is the second leading cause of death worldwide, an average of one in six deaths is due to cancer [1]. Considerable research efforts have been devoted to cancer diagnosis and treatment techniques to lessen its impact on human health. Cancer prediction’s major focus is on cancer susceptibility, recurrence, and prognosis, while the aim of cancer detection is the classification of tumor types and identification of markers for each cancer such that we can build a learning machine to identify specific metastatic tumor type or detect cancer at their earlier stage. With the increased awareness of precision medicine and early detection techniques matured over years of technology development [2–4], including particularly many detection screens achieving a sensitivity around 70–80% [5], the demand for applying novel machine learning methods to discover new biomarkers has become one of the key driving factors in many clinical and translational applications.

Deep learning (DL), a branch of Artificial Intelligence, is a family of multi-layer neural network models that excel at the problem of learning from big data [6]. Similar to other machine learning methods, DL consists of the training step where the estimation of network parameters from a given training dataset is carried out, and the testing step that utilizes the trained network to predict outputs of new input data. The accumulation of whole transcriptomic profiling of tumor samples enabled the pursuit of the DL model for better accuracy and innovative interpretability for cancer type prediction. One prominent resource of cancer transcriptomic profiling is The Cancer Genome Atlas (TCGA) which consists of more than 11,000 tumors from 33 most frequent cancer types [7]. Several DL models have been developed for cancer diagnosis. Ahn, et al., [8] designed a fully connected deep neural network (DNN) trained using a dataset of 6703 tumor and 6402 normal samples, and provided an initial assessment of individual gene’s contribution to the final classification. Lyu et al. [9] and Li et al. [10] extended such an effort to classifying individual tumor types. Li et al. proposed a *k*-nearest neighbors (KNN) algorithm coupled with a genetic algorithm for gene selection and achieved > 90% accuracy for predicting 31 cancer types. Lyu et al. proposed a CNN model with 2D mapping of the gene expression samples as input matrices and achieved > 95% accuracy for all 33 TCGA cancer types. Lyu et al.*,* also provided a data interpretation approach based on Guided Grad-Cam [11]. GeneCT [12] is another attempt which constrains the input genes to 2 categories: oncogenes and tumor suppressors (1076 genes in total) to determine the cancerous status, and transcription factors (1546 genes) to classify samples to the tissue of origin. The paper reported an overall accuracy of 97.8% with the 10-fold cross-validation. Instead of using transcriptomic data, DeepCNA [13], a CNN based classifier, utilized ~ 15,000 samples with copy number aberrations (CNAs) from COSMICS [14] and the HiC data from 2 human cell-lines and achieved an accuracy ~ 60% to discern 25 cancer types. While all these attempts achieved high accuracy to some extent, these methods ignore the existence of tissue of origin within each cancer type. Without removing the influence of normal tissues during cancer classification, the implementation of a data interpretation scheme will unlikely to differentiate tissue-specific genes or cancer-type-specific genes. Thus, it is impossible to perform functional analysis or select biomarkers for cancer detection from such models. Moreover, none of these studies systematically evaluated different CNN model constructions and their impact on the classification accuracy.

In one of our earlier attempts [15], Chen et al constructed an autoencoder system (GSAE) with embedded pathways and functional gene-sets at each input node to reduce the number of weights to be estimated. They applied the GSAE to classify breast cancer subtypes. Here we presented a study of different CNN models constructed for different input data formats. These models systematically interrogate the capacity of the convolution kernels. Utilizing the entire collection of TCGA gene expression data sets, covering all 33 cancer types and nearly 700 normal samples from various tissues of origin, we examined the accuracies of tumor type prediction before and after removing the influence of tissue-specific genes’ expression. In addition, we proposed a unique model interpretation scheme to examine the impact of all key genes that participated in the DL prediction machinery, and we demonstrated the unique characteristics of the proposed CNN models and the feasibility of extracting diagnostic markers for future validation studies.

## Methods

### Datasets

We downloaded pan-cancer RNA-Seq data from The Cancer Genome Atlas (TCGA) [16] by an R/Bioconductor package TCGAbiolinks [17] in December 2018. The dataset contained 10,340 and 713 samples for 33 cancer types and 23 normal tissues, respectively. We represented gene expression by *log*_2_(*FPKM* + 1), where *FPKM* is the number of fragments per kilobase per million mapped reads. Genes with low information burden (mean < 0.5 or st. dev. < 0.8) across all TCGA samples, regardless of their cancer types, were removed. We specifically chose a collection of relative higher overall expression and high variable genes in order to reduce the number of non-informative, or noise-sensitive features, within the dataset. A total of 7091 genes remained after the filtering step. In order to round the input dimension and facilitate the modeling part, nine zeros were added to the gene expressions for having vectors with a length of 7100. We also collected the PAM50 subtypes of 864 breast cancer (BRCA) samples from TCGA [16]. To test the robustness of our models, we added Gaussian noises with zero mean and standard deviations of 0–500% (*k*) of *i*^th^ gene's average expression level (*μ*_*i*_), or *N*(0, *kμ*) to each gene. We set noisy gene expression level to 0 if noise added expression level is less than 0.

### Proposed models

Different CNN models were proposed for cancer type prediction. Each model aims to address a specific aspect of modeling the gene expression data. Few methods were proposed earlier to address input gene order and optimizing the arrangement of genes that leads to the best prediction results in [9] where genes were ordered by their chromosomal positions. In this paper, we kept genes in one preset order but instead, exploit the design of CNN kernels to learn correlations among genes. The other consideration is the depth of CNN. Although deeper CNN models are known to produce more accurate classifications in computer vision [6], several studies have shown that increasing the depth of CNN models on biological data does not always lead to improvement in performance [18]. Here we constrained our designs to include only one layer of convolution. In fact, shallower models are preferred for problems such as cancer type prediction, where there are limited samples relative to the number of parameters. Such shallow models avoid overfitting and also demand fewer resources for training [19, 20]. Based on these two considerations, we presented three different CNN designs next.

#### CNN with vectorized input

**Fig. 1:**
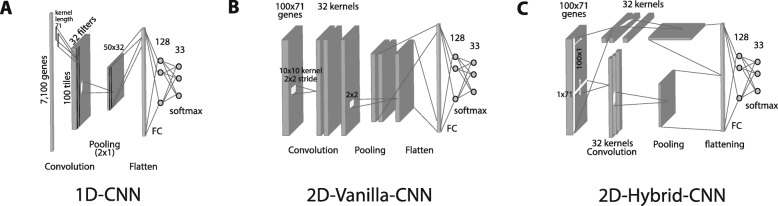
Illustration of three CNN models. **a** 1D-CNN with input as a vector format with 7100 genes. **b** 2D-Vanilla-CNN, with an input reformatted as a 100 × 71 matrix, and one convolution layer. **c** 2D-Hybrid-CNN, similar input as in (**b**) but with two parallel convolution layers, vertical and horizontal, as in (**a**)

This CNN model takes the gene expression as a vector and applies one-dimensional kernels to the input vector. The output of 1-D convolutional layer is then passed to a maxpooling layer, a Fully Connected (FC) layer, and a prediction layer (Fig. 1a). For the sake of simplicity, we call this model *1D-CNN*. The main nuance between the proposed 1D-CNN and other counterpart CNNs for applications such as time series prediction is that the stride of the convolution is the same as the length of kernel size. As a matter of fact, in some applications, 1D CNN is harnessed to capture temporal relationships between adjacent values in the input. However, in our case, since we are not confident that there are correlations among neighboring gene expression values in the input vector, we choose the stride of CNN as big as the kernel size to capture only the global features associated with this kernel.

#### CNN with matrix input

The second CNN model follows the most commonly practiced types of CNN applications in computer vision where the input has a 2-D format like an image. This CNN includes 2D kernels to extract local features in the input Fig. 1b. Similar to [9], we reshaped the input gene expression into the 2D space without any specific arrangement to construct an image-like input before feeding it to the 2D CNN. The 2D CNN includes the convolutional layer with the 2D kernel, a maxpooling layer, an FC layer, and a prediction layer. For convenience, we term this model as the *2D-Vanilla-CNN*.

#### CNN with matrix input and 1D kernels

The third model is the *2D-Hybrid-CNN*, which is inspired by the parallel towers in the Resnet modules [21] and the simplicity of our proposed 1D-CNN. It is proposed to take advantage of having 2-D inputs with simple 1D convolution operations. In this model, as can be seen in Fig. 1c, two 1D-kernels slide over the inputs, where one with the size of a row slides vertically and the other one with the size of a column slides horizontally across the 2D input. The outputs of two 1D-kernels are then passed through a maxpooling layer before being concatenated and fed into the FC and prediction layers. As in the Resnet modules, we believe this design can capture more global unstructured features in the input gene expression.

#### Implementation of 2D-3Layer-CNN

We implemented the model proposed in [9] with all details in Keras DL platform and named it 2D-3Layer-CNN in order to have a fair side-by-side comparison between CNN models developed in this paper. This model contains three convolution modules which in each one Batch Normalization, Activation Function (AF), and Maxpooling are connected in a cascade manner. The output of the last convolution module is fed into two FC layers and finally softmax layer is used for predicting 33 different cancer types.

### CNN model interpretation

We utilized the guided gradient saliency visualization method provided by the Keras visualization package keras-vis [22]. This method calculates the output gradient classes with respect to a small change in gene expressions. The positive values of these changes prime us the importance of those gene expressions in the inputs [23]. In the saliency map generation step, each sample was fed into the model to construct an interpretation map. We then summarized each cancer type as well as for the normal samples by averaging across all samples of the group and constructed a gene-effect matrix of 7091 × 34 (33 cancer type and one normal class) that contains gene-effect scores with a range of [0, 1] with 1 s have maximum effect and 0 to no effect. A gene with a gene-effect score greater than 0.5 was defined as a marker gene for a given cancer.

## Results

### Model construction, hyperparameter selection and training

All of the three models were implemented by Keras [24] DL platform. All of the codes can be found at https://github.com/chenlabgccri/CancerTypePrediction. The input for 1D-CNN (Fig. 1a) is a 1D vector following gene symbol’s alphabetic order, while inputs for 2D-Vanilla-CNN and 2D-Hybrid-CNN (Fig. 1b,c) models were reshaped to 100 rows by 71 columns matrix. Four of the key hyperparameters known as the number and size of kernels, the stride of kernels, and the number of nodes in the FC layer were tuned by the Grid search method provided in [25]. The Tables 1 and 2 show all sets of parameters were chosen for 1D-CNN and 2D-Vanilla-CNN models respectively, and their statistical measures on train and test pools. In addition, Categorical Cross Entropy as the loss function, Categorical accuracy as training metric and the Adam optimizer were selected for all 3 CNN models. The epoch and batch size were chosen as 50 and 128, respectively, with the early stopping set with patience = 4 to stop the learning in the case that categorical accuracy did not improve in four consecutive epochs. Finally, ReLU was used as the AF and softmax as the prediction layer at the final layer for all the models.

**Table 1:** Different hyperparameter settings for 1D-CNN model based on the trained and tested statistical measures. The final selected parameters are highlighted

Hyperparameters			Loss			
dense layer size	filter	kernel	mean train_score	stdev train_score	mean test_score	stdev test_score
64	(1, 50)	8	0.069	0.031	0.167	0.023
64	(1, 50)	16	0.037	0.013	0.140	0.007
64	(1, 50)	32	0.023	0.003	0.132	0.006
64	(1, 50)	64	0.013	0.002	0.128	0.006
128	(1, 50)	8	0.032	0.008	0.147	0.006
128	(1, 50)	16	0.027	0.014	0.138	0.014
128	(1, 50)	32	0.011	0.003	0.121	0.009
128	(1, 50)	64	0.004	0.001	0.126	0.012
512	(1, 50)	8	0.009	0.000	0.138	0.008
512	(1, 50)	16	0.006	0.001	0.127	0.003
512	(1, 50)	32	0.124	0.179	0.265	0.160
512	(1, 50)	64	0.003	0.002	0.125	0.008
64	(1, 71)	8	0.072	0.009	0.177	0.009
64	(1, 71)	16	0.044	0.009	0.149	0.006
64	(1, 71)	32	0.036	0.011	0.135	0.009
64	(1, 71)	64	0.016	0.004	0.124	0.012
128	(1, 71)	8	0.046	0.007	0.154	0.015
128	(1, 71)	16	0.027	0.006	0.135	0.015
**128**	**(1, 71)**	**32**	**0.014**	**0.002**	**0.129**	**0.016**
128	(1, 71)	64	0.008	0.001	0.119	0.003
512	(1, 71)	8	0.023	0.018	0.152	0.023
512	(1, 71)	16	0.009	0.008	0.132	0.017
512	(1, 71)	32	0.004	0.002	0.123	0.008
512	(1, 71)	64	0.011	0.016	0.134	0.015
64	(1, 100)	8	0.088	0.010	0.172	0.015
64	(1, 100)	16	0.066	0.014	0.162	0.009
64	(1, 100)	32	0.037	0.007	0.132	0.009
64	(1, 100)	64	0.024	0.009	0.128	0.013
128	(1, 100)	8	0.058	0.001	0.164	0.009
128	(1, 100)	16	0.031	0.008	0.144	0.014
**128**	**(1, 100)**	**32**	**0.019**	**0.004**	**0.128**	**0.008**
128	(1, 100)	64	0.016	0.010	0.137	0.027
512	(1, 100)	8	0.031	0.013	0.155	0.014
512	(1, 100)	16	0.009	0.001	0.135	0.009

**Table 2:** Different hyperparameter settings for 2D-Vanilla-CNN model based on the trained and tested statistical measures. The final selected parameters are highlighted

Hyperparameters				Loss			
dense layer size	filter	kernel	stride	mean train_score	stdev train_score	mean test_score	stdev test_score
128	32	(7, 7)	(1, 1)	20.999	18.228	21.281	14.904
128	32	(7, 7)	(2, 2)	0.005	0.002	0.192	0.022
128	32	(10, 10)	(1, 1)	21.398	18.582	21.771	15.298
**128**	**32**	**(10, 10)**	**(2, 2)**	**0.009**	**0.003**	**0.187**	**0.008**
128	32	(20, 20)	(1, 1)	0.027	0.004	0.202	0.029
128	32	(20, 20)	(2, 2)	0.043	0.011	0.206	0.009
128	64	(7, 7)	(1, 1)	10.213	17.688	10.566	14.618
128	64	(7, 7)	(2, 2)	0.004	0.001	0.187	0.018
128	64	(10, 10)	(1, 1)	31.430	1.149	31.675	1.019
128	64	(10, 10)	(2, 2)	0.012	0.006	0.177	0.014
128	64	(20, 20)	(1, 1)	12.020	18.052	12.149	14.818
128	64	(20, 20)	(2, 2)	0.055	0.016	0.204	0.020
512	32	(7, 7)	(1, 1)	21.245	18.419	21.175	14.815
512	32	(7, 7)	(2, 2)	10.944	18.953	11.022	15.306
512	32	(10, 10)	(1, 1)	10.964	18.987	11.148	15.482
512	32	(10, 10)	(2, 2)	0.003	0.001	0.213	0.025
512	32	(20, 20)	(1, 1)	10.988	19.002	11.132	15.436
512	32	(20, 20)	(2, 2)	1.110	1.849	1.271	1.397
512	64	(7, 7)	(1, 1)	31.430	1.149	31.675	1.019
512	64	(7, 7)	(2, 2)	10.213	17.688	10.560	14.622
512	64	(10, 10)	(1, 1)	31.497	1.211	31.648	1.087
512	64	(10, 10)	(2, 2)	20.628	17.858	20.481	14.363
512	64	(20, 20)	(1, 1)	11.299	16.825	11.562	13.969
512	64	(20, 20)	(2, 2)	12.020	18.046	12.152	14.776

**Fig. 2:**
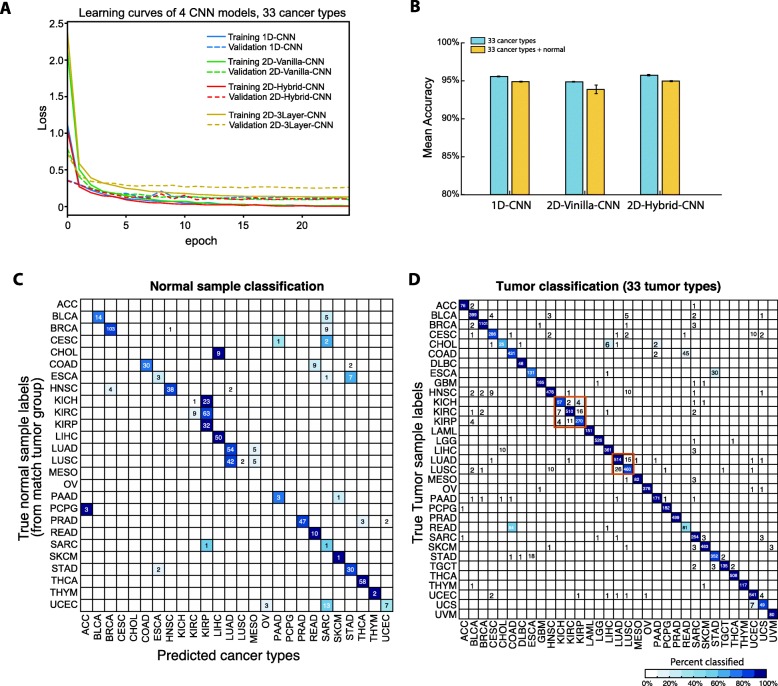
Cancer type prediction performance of three CNN models trained with tumor samples only. **a** Learning curves for all three CNN models. **b** Micro-averaged accuracy of three CNN models when trained with only tumor samples (light blue) from 33 tumor types, and with tumors and normal samples together (light brown). **c** Confusion matrix of normal samples prediction from 1D-CNN model trained with 33 tumor types only. **d** Confusion matrix of the 1D-CNN model on all 33 tumor types

All three CNN models were trained with all 10,340 tumor samples initially. To evaluate the training procedure and their robustness against overfitting, we examined loss functions for 3 models Fig. 2a using 80–20% splitting for training and validation, and we observed converges to ~ 0 loss after 10 epochs (where validation’s loss at about 0.10 with no obvious overfitting). The model in [9] was trained and tested with the same procedure. As can be seen in Fig. 2a, the convergence of this model is slower than all proposed three models in this paper.

In order to avoid the bias impacted by the stochastic dependency nature of neural networks during training, the 5-fold cross validation was repeated six times (due to the time constraint) and their mean and standard deviation of the classification accuracy were reported for all models. Figure 2b (light blue bars) showed classification accuracy at 95.5 ± 0.1%, 94.87 ± 0.04%, 95.7 ± 0.1% for 1D-CNN, 2D-Vanilla-CNN and 2D-Hybrid-CNN, respectively.

### Assessing the impact of tissue-specific features on cancer type prediction

Considering the tissues of origin when classify tumor samples, previous studies either omitting this important factor by only training the DL machine with tumor samples and then looking for cancer driver genes [9], or training two models: with only cancer associated genes (tumor DL model) or transcription factors (normal DL model) [10]. To observe the influence of tissues of origin with DL model trained with tumor sample only, we fed all 713 normal samples that matched 23 TCGA cancer types into 1D-CNN model trained on 33 cancer types in the previous section. As is shown in Fig. 2c**,** 19 of 23 normal classes are classified into their corresponding cancer type, where normal samples from kidney (KICH, KIRC and KIRP), liver (CHOL and LIHC), lung (LUAD and LUSC) or digestive system (ESCA and STAD) are clearly grouped together, indicating a strong possibility that DL machine was partially trained to recognize tissues of origin. When we examined the classification results of tumor samples (Fig. 2d), the major classification errors are also within the kidney, lung (both boxed in Fig. 2d), colon and rectum adenocarcinomas.

### Predicting cancer types without the influence of tissue of origin

**Fig. 3:**
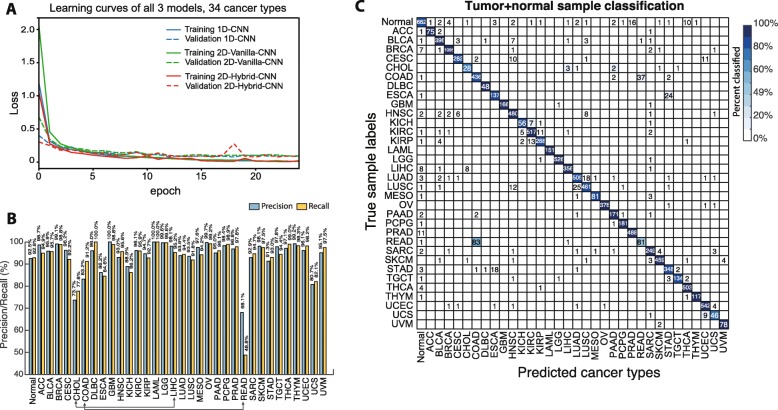
Cancer type prediction performance of three CNN models trained with combined tumor and normal samples. **a** Learning curves for all three CNN models trained with combined tumor and normal samples. **b** Precision (light blue) and recall (light brown) of 1D-CNN model when trained with combined tumor normal samples. **c** Confusion matrix of all sample prediction from 1D-CNN model trained with 33 tumor types + normal

In order to take into account the impact of tissue of origin in the model, we introduce a new label in the prediction layer where it takes all normal samples (regardless of their original tissue type designation). The 34th node in the prediction layer is responsible to remove the trace of tissue of origins from cancer samples, with the intention of achieving a robust cancer type prediction. All three models were re-trained with 33 nodes for tumor classes plus one node for normal samples (labeled as “Normal”) with the same architectures correspondingly. Similar to model training with 33 cancer-types only, we had a consistent learning curve (Fig. 3a) using 80–20% splitting for training and validation, and converged to ~ 0 loss after 10 epochs without obvious overfitting. As shown in Fig. 2b (brown bars), we achieved the overall accuracies 94.9 ± 0.1%, 93.9 ± 0.6%, 95.0 ± 0.1% for 1D-CNN, 2D-Vanilla-CNN and 2D-Hybrid-CNN, respectively, slightly lower than 33 cancer only training, due to the introduction of normal samples (Precision at 92.5%, Fig. 3b).

Further evaluation of micro-averaged precision-recall statistics of 1D-CNN model with 34 output nodes yielded some interesting observations (Fig. 3b). The DL machine has a large discrepancy in the precision-recall value of tumor type READ. This is due to the large number READ (rectum adenocarcinoma, 83) samples misclassified into COAD (colon adenocarcinoma), causing much lower recall level (48.8%) (Fig. 3c), while 37 COAD samples are misclassified into the READ type. Cholangiocarcinoma (CHOL) has only 36 tumor samples total but a large fraction misclassified into hepatocellular carcinoma (LIHC, 3 samples (~ 9%)) and Pancreatic Adenocarcinoma (PAAD, 2 samples). Cholangiocarcinoma is a bile duct cancer, and specifically the distal region (extrahepatic cholangiocarcinoma) is made up of the common bile duct that passes through the pancreas, thus potentially the cause of misclassification. We have attempted to train with a separated kidney normal tissue group with no clear improvement (data not shown). Evidently, more normal samples per tumor group could further improve the performance.

### Interpretation of the 1D-CNN model to investigate cancer marker genes

We systematically investigated the 1D-CNN model to understand how the model predicted cancer types with the aim to identify cancer marker genes. The interpretation was accomplished by generating the saliency map (see Methods Section) of 1D-CNN model.

#### Interpretation of the 1D-CNN model to investigate cancer marker genes

**Fig. 4:**
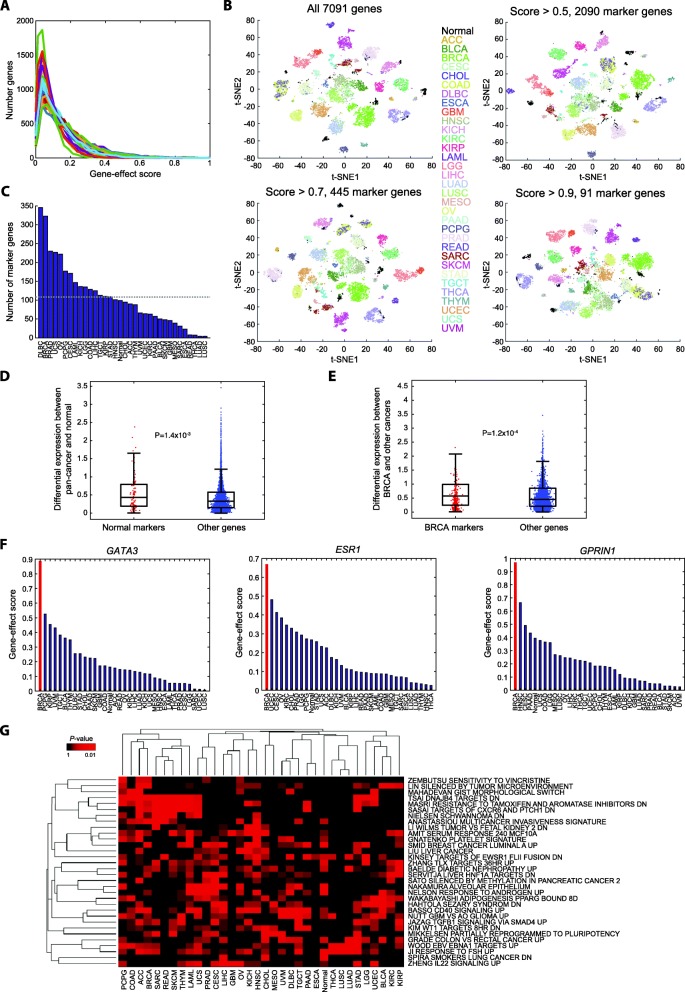
Interpretation of the 1D-CNN model. **a** Distributions of gene-effect scores for individual cancer and normal classes. Colors correspond to cancer types denoted in Fig. 4b. **b** t-SNE plots of pan-cancer and normal samples by expression of marker genes identified using different thresholds. **c** Marker genes identified in each class with a criterion of gene-effect score > 0.5. The dashed line denotes the average number of marker genes identified across 34 classes. **d**-**e** Differential expression of marker genes and other genes between sample classes. Here differential expression is presented by an absolute difference between a class (normal or BRCA) and all other samples in log_2_(FPKM+ 1). **f** Pan-classes gene-effect scores of three marker genes of BRCA. **g** Functions associated with marker genes identified in each class

We first examined the distribution of gene-effect scores of saliency maps for all cancer types, and generally they followed the power law (Fig. 4a). We set criteria on the gene-effect scores to identify marker genes (see Methods). t-SNE plots on expression data of selected marker genes confirmed that the identified markers preserved the differences among classes even when stringent thresholds were set (scores > 0.5 and > 0.9 yield 2090 and 91 unique marker genes, respectively; Fig. 4b). To include more potential cancer markers into the investigation, we used the threshold of 0.5 for subsequent analyses. We obtained a total of 3683 markers (2090 unique genes) for all the 34 classes with a minimum of 4 markers to a maximum of 346 (Fig. 4c), or average ~ 108 markers per cancer type. Diffuse large B-cell lymphoma (DLBC), breast invasive carcinoma (BRCA), and prostate adenocarcinoma (PRAD) were found with the most markers (346, 323, and 230, respectively). Interestingly, the cancers that our model tended to confuse, such as lung cancers (adenocarcinoma [LUAD] and squamous cell carcinoma [LUSC]) and rectum adenocarcinoma (READ), had a much smaller number of markers (4, 4, and 8, respectively). The finding suggested our model’s low confidence in classifying cancer types with few marker genes and the requirement of additional modes of genomics profiles (methylation, etc.) to further discriminate cancer types within the same tissue of origin.

#### Discrimination capability of marker genes

We investigated whether simple linear-like differential expression between classes underlying the capacity of these marker genes. The 99 marker genes with a gene-effect score > 0.5 obtained from the normal class indeed had significantly larger differences in the expressional level between pan-cancers and normal samples than other genes (*t*-test *P* = 1.4 × 10^− 3^; Fig. 4d), though the differences were moderate in magnitude (mean, 0.55 vs. 0.43). Taking BRCA as a demonstrating example, 323 BRCA markers had a larger differential expression between BRCA and other cancer samples than 6768 non-marker genes (*P* = 1.2 × 10^−4^; Fig. 4e). The phenomenon held for all markers of any cancer types (*P* = 1.6 × 10^− 47^). Taken together, our model indeed identified genes with differential expression between classes.

#### Marker genes in the breast cancer group

We further examined a well-studied cancer type, BRCA, as a demonstrating example to the marker genes identified by our model. BRCA had 323 marker genes (gene-effect score > 0.5). Well-known specific markers of BRCA, such as *GATA3* [26] and *ESR1* [27] were ranked at the 13th and 98th among all genes. Their classifying capability was predominantly in BRCA (gene-effect scores, 0.89 and 0.67; Fig. 4f). Also, we identified other promising novel markers of BRCA, such as *GPRIN1* (the top marker gene with a score of 0.97; Fig. 4f), *EFNB1* (2nd, score = 0.94), and *FABP4* (3rd, score = 0.92), that warrant further investigations.

#### Biological functions of marker genes

To understand biological functions underlying cancer classification, we performed a functional annotation analysis on marker genes of each cancer type or normal. Each set of marker genes were systematically tested for enrichment in a chemical and genetic perturbation signature (the CGP collection) curated by the Molecular Signature Database (MSigDB) [28, 29]. With a criterion on one-tailed Fisher’s exact test at *P* < 0.001, we identified a total of 32 associated functions among the 34 classes (Fig. 4g). Among the top function-class pairs we identified several known cancer functions. For instance, a signature identified from a soft tissue cancer, ‘NIELSEN SCHWANNOMA DN’ [30], was significantly associated with markers of sarcoma (SARC) (top 2nd significant function-class pair; *P* = 3.3 × 10^− 5^). Also, marker genes of prostate adenocarcinoma (PRAD) were associated with a signature of androgen response, ‘NELSON RESPONSE TO ANDROGEN UP’ [31](*P* = 5.8 × 10^− 4^). We also identified several novel marker functions of cancers, such as ‘BASSO CD40 SIGNALING UP’ in testicular germ cell tumor (TGCT) (1st; *P* = 2.0 × 10^− 5^), and ‘WAKABAYASHI ADIPOGENESIS PPARG BOUND 8D’ in bladder urothelial carcinoma (BLCA) (3rd, *P* = 4.1 × 10^− 5^). Overall, functional annotation analysis validated what we expected and potentially revealed several novel mechanisms through the CNN model interpretation. However, much of the functional interrogation remained to be further studied.

### Breast cancer subtype prediction

While predicting cancers from different anatomic sites may be relatively straightforward, predicting cancer subtypes, such as breast cancer, is an ongoing research topic. Breast cancer is divided into four subtypes known as luminal (A&B), HER2 positive and basal (often triple-negative breast cancers (TNBC)) breast cancers [32]. In order to accomplish this, we further trained 1D-CNN model with all breast cancer samples from four different subtypes plus the normal breast cancer and set the prediction layer to 5 nodes. To further simplify the 1D-CNN, the fully connected layer with 128 nodes was removed. After training, we achieved an average precision of 88.3% (details in Table 3). The misclassification was mainly between luminal A & B classes since they are two inherently similar tumor subtypes; or in the Her2 class due to limited information captured by expression profiles since it is defined as the gain in DNA copy number and/or over-expression of the ERBB2 gene.

**Table 3:** Breast cancer subtype classification using 1D-CNN model

Class name	Precision	Recall	F1-score	Number of samples
Basal	0.973	0.980	0.976	147
Her2	0.829	0.853	0.841	68
Luminal A	0.894	0.927	0.910	437
Luminal B	0.810	0.780	0.795	186
Normal	0.857	0.462	0.600	26
Avg/Total	0.883	0.884	0.882	864

## Discussion

There were several critical issues that this paper addressed to improve the accuracy of our prediction and interpretation. Specifically, three CNN architectures were proposed to investigate an appropriate architecture for unstructured gene expressions for predicting cancer types. As is shown in Fig. 2b, 1D-CNN and 2D-Hybrid-CNN achieved comparable accuracy (95.7%), which improves the result (95.6%) slightly in [9]. Note that 2D-Vanilla-CNN contains only one layer and 32 kernels, whereas the 2D-3Layer-CNN consists of multiple DL modules, a much more complex architecture. In addition to what we summarized in Table 4 where the number of parameters for each model, loss function value after training and testing, and execution time examples, we note several underlying design facts behind each proposed model.

- The *1D-CNN* is significantly simpler than the other models proposed in the literature. It does not require inputs to be arranged in a particular order and it has only one convolutional layer. This much-simplified design induces a significant reduction in the number of hyperparameters (from 26 million to ~ 200 thousand) to be estimated during training. This is highly desirable in the DL applications in genomic studies due to the difficulty and the high cost of collecting large genomic data.
- The *2D-Vanilla-CNN* has around one million hyperparameters which are significantly more than those of the 1D-CNN. The model became more difficult to converge when the stride of the kernel was selected to be 1 × 1. Also, by sliding two separate convolutions kernels over the two orthogonal dimensions, it improved the accuracy due to the ability to capture more global features.

**Table 4:** Hyperparameters and training time of CNN models

		Training		Testing		
DL model^a^	Number of parameters	Loss	Accuracy	Loss	Accuracy^b^	Time^c^ (seconds)
1D-CNN	211,489	0.01	0.9971	0.1769	0.9567	80.3
2D-Vanilla-CNN	1,420,737	0.007	0.9981	0.1778	0.9557	94
2D-Hybrid-CNN	362,177	0.0149	0.996	0.1586	0.9582	80.8
2D-3Layer-CNN	26,211,233	0.5149	0.9654	0.6875	0.9184	214.6
2D-3Layer-CNN (with patience = 10)		0.1976	0.9869	0.3914	0.9419	379.17

^a^Early stopping is used for all models (all with patience = 4, except for the last model)

^b^Results of 5-fold cross-validations

^c^All models were trained using a Linux server with Xeon 8176 CPU @2.1GHz, with 4 × 28 cores

While 2D-Hybrid-CNN may provide a slight advantage in terms of the averaged classification accuracy (Fig. 2b), it has two times more hyperparameters and thus a higher computation burden compared with the 1D-CNN model. Therefore, we focused on the 1D-CNN model in most of our subsequent analysis.

**Fig. 5:**
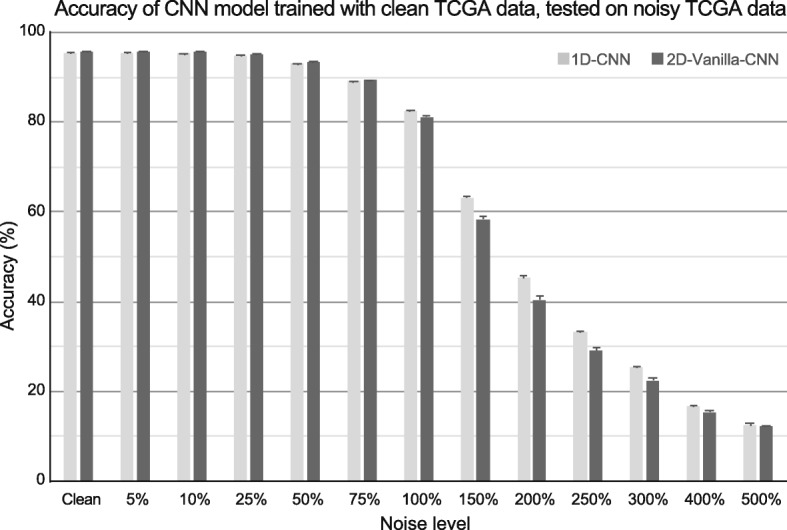
CNN models testing on noisy data. Classification accuracy on TCGA data with different additive Gaussian noise added. Both classifiers were trained with original TCGA data and tested on TCGA data + Gaussian noise

2D-Vanilla-CNN had similar accuracy comparing to 1D-CNN, but had almost 5x more hyperparameters to train. In order to investigate the robustness of proposed models in the presence of noise, both CNN models were tested with data added with different levels of noise as explained in the Methods section. In Fig. 5, the 5-fold cross-validation accuracy of 1D-CNN and 2D-Vanilla-CNN while tested on different ratios of noise are represented. As it was shown, the performance of both models was extremely robust until the noise ratio reached 75% and then it gradually dropped. Although both models had almost equal performance results until 75% noise ratio, 1D-CNN outperformed 2D-Vanilla-CNN in noise ratios above 75%. Thus, we conclude 1D-CNN has more stable performance encountering unwanted noise compared to other models.

We chose to combine tumor samples plus normal samples together to train a DL model with 34 nodes in the prediction layer such that we can eliminate the influence of tissue origin in cancer type prediction. The model not only achieved a good precision in predicting normal tissues (92.5% precision) but made few mistakes in distinguishing cancer types within the same tissue origin; examples include KICH, KIRC, and KIRP, all of which are kidney cancers, where only 2 normal samples were classified into cancer groups (out of 128 normal kidney samples, Fig. 3c). We will continue our work to resolve this issue by introducing yet another rich source of transcriptomic data from GTEx collection [33]. Furthermore, as suggested by previous studies [13, 15, 34–38], we may incorporate additional genome-wide profiling information, such as DNA mutation, copy number variation, and DNA methylation as additional input matrices to enrich the complexity for model training, and thus to improve the classification accuracy.

Our unique interpretation of the CNN for genomic data has shown its utility when we examined the gene-effect scores. While some of these differences are modest in magnitude, our DL machine had no trouble to classify tumors into their correct subtypes, indicating a simple linear classifier (i.e., expression high vs. low) might not explain the complexity of our CNN. In this sense, our CNN model had the benefit of capturing high-order interactions among these genes to make accurate predictions.

## Conclusions

Taken together, we have presented three unique CNN architectures that take high dimension gene expression inputs and perform cancer type prediction while considering their tissue of origin. Our model achieved an equivalent 95.7% prediction accuracy comparing to earlier published studies, however with a drastically simplified CNN construction and with a reduced influrence of the tissue origin. This allows us to perform a model interpretation of our CNN to elucidate cancer markers for each cancer type, with hope in future refinement that will lead to markers for earlier cancer detection.

## Data Availability

The dataset supporting the conclusions of this article is included within the article.

## Abbreviations

ACC: Adrenocortical cancer
BLCA: Bladder urothelial carcinoma
BRCA: Breast invasive carcinoma
CESC: Cervical and endocervical cancer
CHOL: Cholangiocarcinoma
CNN: Convolutional neural network
COAD: Colon adenocarcinoma
DL: Deep learning
DLBC: Diffuse large B-cell lymphoma
ESCA: Esophageal carcinoma
GBM: Glioblastoma multiforme
HNSC: Head and neck squamous cell carcinoma
KICH: Kidney chromophobe
KIRC: Kidney clear cell carcinoma
KIRP: Kidney papillary cell carcinoma
LAML: Acute myeloid leukemia
LGG: Lower grade glioma
LIHC: Liver hepatocellular carcinoma
LUAD: Lung adenocarcinoma
LUSC: Lung squamous cell carcinoma
MESO: Mesothelioma
OV: ovarian serous cystadenocarcinoma
*P*: *P*-value
PAAD: Pancreatic Adenocarcinoma
PCPG: Pheochromocytoma and paraganglioma
PRAD: Prostate adenocarcinoma
READ: Rectum adenocarcinoma
SARC: Sarcoma
SKCM: Skin cutaneous melanoma
STAD: Stomach adenocarcinoma
TCGA: The Cancer Genome Atlas
TGCT: Testicular germ cell tumor
THCA: Thyroid carcinoma
THYM: Thymoma
UCEC: Uterine corpus endometrioid carcinoma
UCS: Uterine carcinosarcoma
UVM: Uveal melanoma
